# Adverse Weather Target Detection Algorithm Based on Adaptive Color Levels and Improved YOLOv5

**DOI:** 10.3390/s22218577

**Published:** 2022-11-07

**Authors:** Jiale Yao, Xiangsuo Fan, Bing Li, Wenlin Qin

**Affiliations:** 1 College of Automation, Guangxi University of Science and Technology, Liuzhou 545006, China; 2 School of Resources and Environment, University of Electronic Science and Technology of China, Chengdu 611731, China; 3 Guangxi Collaborative Innovation Centre for Earthmoving Machinery, Guangxi University of Science and Technology, Liuzhou 545006, China

**Keywords:** adverse weather, adaptive color levels, YOLOv5, transformer, CBAM

## Abstract

With the continuous development of artificial intelligence and computer vision technology, autonomous vehicles have developed rapidly. Although self-driving vehicles have achieved good results in normal environments, driving in adverse weather can still pose a challenge to driving safety. To improve the detection ability of self-driving vehicles in harsh environments, we first construct a new color levels offset compensation model to perform adaptive color levels correction on images, which can effectively improve the clarity of targets in adverse weather and facilitate the detection and recognition of targets. Then, we compare several common one-stage target detection algorithms and improve on the best-performing YOLOv5 algorithm. We optimize the parameters of the Backbone of the YOLOv5 algorithm by increasing the number of model parameters and incorporating the Transformer and CBAM into the YOLOv5 algorithm. At the same time, we use the loss function of EIOU to replace the loss function of the original CIOU. Finally, through the ablation experiment comparison, the improved algorithm improves the detection rate of the targets, with the mAP reaching 94.7% and the FPS being 199.86.

## 1. Introduction

With the continuous development of artificial intelligence, self-driving vehicles have become a popular topic in people’s lives. People are increasingly concerned about the safety of vehicle driving, especially in adverse weather such as rain, fog, snow and sandstorms [1]. Due to the low visibility, the external environment hinders the vehicle detection system, which seriously affects the performance of computer vision algorithms and has an impact on driving safety. At the same time, the diverse traffic environment and rich and diverse traffic flow also pose challenges to computer vision algorithms. Therefore, the detection of road targets by self-driving vehicles in adverse weather has become a popular topic in current research. In self-driving vehicles, the vision system plays a major role in ensuring the smooth driving and personnel safety of the vehicle. Accurate perception of the external environment and obtaining information about the vehicle are of great significance for safe vehicle driving. Image processing and target detection algorithms, as a crucial topic in computer vision, can detect external information accurately and in real-time, which is the primary requirement in the real world of self-driving vehicles [2,3].

Traditional target detection algorithms generally extract the gradient histogram information of the image first and then classify and detect the target by the SVM of the machine learning algorithm. The disadvantage of such algorithms is that they require manual extraction of image features, which is a complex process and which has low detection accuracy. Deep learning-based target detection algorithms do not require manual feature extraction and can be trained to extract features through neural networks autonomously. The current popular target detection algorithms are two-stage algorithms and single-stage algorithms. Two-stage algorithms based on a candidate frame have high detection accuracy but slow detection speed, such as Fast R-CNN, Faster R-CNN, Mask R-CNN, etc. The single-stage algorithms, such as SSD and YOLO series algorithms, are faster but less accurate due to the end-to-end training method. Currently, YOLOv5 is increasingly used to study specific topics, and the improved algorithm has good results [4,5].

It is necessary to study the previous results for this project. Li summarizes the development and status of target detection algorithms [5], which gives us a deeper understanding of target detection algorithms. Ting et al. merged the feature extraction process with the Ghostbottlenet algorithm to improve the accuracy of the YOLOv5 algorithm to solve the problem of insufficient feature extraction in current ship identification methods. The final mAP was as high as 99.85%, which is a 2.18% improvement over the original network [6]. Zhu et al. used the attention mechanism and ECA-Net used to improve YOLOv5 to highlight information that contributes to boulder detection. Experimental results showed that the performance accuracy of the improved YOLOv5 improved by 3.4% [7]. Zhu et al. added a transformer encoding module, CBAM and some specialized tricks to YOLOv5 for target object detection in UAV capture scenes. The mAP was improved by 7% compared to the baseline model [8]. Shi et al. introduced a method of channel attention mechanism in YOLOv5 to solve the problem of insufficient information on tiny target features. Experimental results show that the improved model has improved in terms of detection accuracy and recall compared with the original YOLOv5 algorithm [9]. Zhou et al. improved the YOLOv5s algorithm to achieve real-time detection of unmanned fishing speedboats near ships ahead. They optimized the loss function by reclustering the targets with K-means on the data input side and expanding the acceptance domain region on the output side. The results show that the improved model achieves 98.6% mAP, which is 4.4% better than before the improvement [10]. Xie et al. proposed a novel lightweight end-to-end target detection method and fused the framework with an attention module into the YOLOv5 algorithm for ship detection. Experimental results on remote sensing images from synthetic aperture radar (SAR) show that the method has significant improvements in efficiency and performance [11]. Zhu et al. designed a bidirectional feature pyramid network for feature detection fusion so that feature layers at different scales can better learn the weight distribution and enhance the fusion capability [12]. Zhang et al. used the improved network with the transformer for detecting multi-scale targets, ultimately improving the detection accuracy by 1.9% [13]. Fu et al. incorporated CBAM into the target detection algorithm and used it for marine target detection. The method can increase the weights of useful features while suppressing the weights of invalid features as a way to improve detection accuracy. The experimental results show that the improved algorithm has higher detection accuracy than the original algorithm while providing better detection results for small targets, multiple targets and overlapping targets [14].

To enhance the robustness of the model so that the model can accurately recognize objects of all sizes is also one of our research directions. Studying the problem of multi-scale target detection can help in the recognition rate. Walambe et al. proposed an implementation of integrated transfer learning to improve the performance of the underlying multi-scale target detection model in UAV images and combined it with a voting strategy to recognize targets. The method helps in the recognition of multiscale targets [15]. A framework was proposed by Khan et al. for solving the recognition problem of high-resolution satellite images. Khan et al. proposed a framework for solving the recognition problem of high-resolution satellite images. The framework consists of two stages. The first stage generates multi-scale object proposals and the second stage experimental results, which show that this method outperforms other methods in the detection of multi-scale objects [16]. Cheng et al. investigated a SAS-YOLOv3-Tiny algorithm that combines depthwise separable convolution and a channel attention mechanism for solving the detection problem of multi-scale safety helmets. This method significantly improves all performance metrics compared to the original algorithm [17]. Gao studied a DSS algorithm that dynamically selects samples using the shape and size of the target. DSS brings to ATSS about 0.7% mAP improvement on the MS COCO dataset [18]. A parallel-assisted multiscale feature enhancement module MFEM was used to solve the parallel multiscale small target detection problem by Liang et al. It was demonstrated experimentally on MS COCO that the improved FE-RetinaNet algorithm achieved a detection accuracy (mAP) improvement of 1.8% on MS COCO [19].

The study of current image processing methods in adverse weather has inspired image preprocessing work in this paper. He et al. used a dark channel prior method to directly estimate the thickness of the haze and recover it into a high-quality clear image with good results [20]. Zhu et al. proposed a color attenuation prior method that can effectively remove the blurred background of a single input image with high efficiency [21]. By analyzing a large number of foggy images, it was found that the fog concentration varies with the depth of field, and the higher the fog concentration, the greater the depth of field, and the greater the difference in brightness and saturation of the image. Based on this, a color decay a priori fog removal algorithm was designed. The experimental results show that the method outperforms existing fog removal algorithms in terms of efficiency and fog removal effect. Tan et al. used the Maximizing Contrast method for automatic image defogging and achieved a better result [22]. The chromaticity inconsistency method of Ancuti et al. is simpler and performs faster than existing strategies while producing comparable or even better results [23]. Manjunath et al. used the Color Attenuation Prior method for removing the haze background from a single image. Experimental results show that the proposed method outperforms the state-of-the-art haze removal algorithms in terms of both efficiency and defogging effect [24]. Katiyar et al. used Color Attenuation Prior and Multi-Scale Fusion for haze removal from a single image. The experimental results show that the method has good results [25]. Li et al. proposed an image deblurring model for integrated deblurring, and the method is outstanding for object detection on blurred images [26]. Cai et al. designed an end-to-end system for individual image deblurring, which improves the quality of recovered haze-free images. Experiments have shown that the method has excellent performance and is simple yet efficient [27].

For this paper, the main contributions are as follows.

(1) To address the problem of difficulty in recognition of autonomous vehicles in adverse weather, we propose a bias compensation model to perform adaptive color levels correction on images, which can effectively improve the sharpness of adverse weather images. Since adverse weather images have different mean values, after calculating the image mean values, the images of different categories of weather are classified and assigned to different channels for filtering to improve clarity of the input images.
(2) In order to have a better recognition rate in adverse weather, we choose to improve on the YOLOv5 algorithm. By incorporating the transformer, it makes a smoother connection from low-level features to high-level features, further integrates the features extracted by the backbone, and focuses feature information. At the same time, CBAM is added between Neck and Head to further gather key information and solve the problem of invalid features affecting recognition accuracy. Finally, we adopt the activation function of EIOU to replace the original CIOU activation function to further improve the accuracy of the YOLOv5 model. The improved algorithm is found to have a significant improvement over the previous one through ablation experiments.
(3) In this paper, we use SSD, YOLOv3-tiny, YOLOv4-tiny, YOLOv5 and the improved YOLOv5 algorithm to test under the same dataset. The results show that the improved algorithm proposed in this paper outperforms the YOLOv5 baseline algorithm in both recognition accuracy and recognition speed, which proves the effectiveness and advancement of the algorithm in this paper.

## 2. Dataset Processing

### 2.1. Image Acquisition and Enhancement

Although there are various datasets in the field of autonomous driving, such as KITTI, ApolloScape, BDD100K, etc., it is difficult for these datasets to meet the conditions of different severe weather, different resolutions, different target scales and the different number of targets at the same time [15]. In contrast, the DAWN [1] dataset is more suitable for our needs in terms of severe environment diversity and sample realism; thus, we finally based our study on the open-source dataset DAWN.

**Figure 1:**
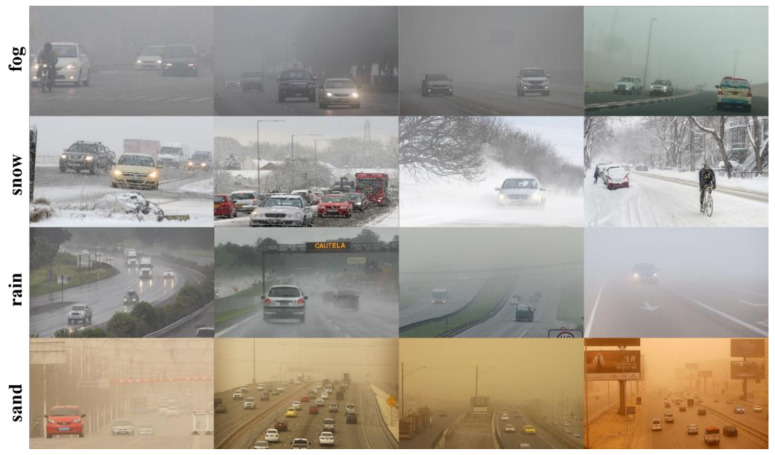
Schematic diagram of the adverse weather dataset.

The DAWN dataset was captured and collected from people, cars, buses, trucks, motorcycles, and bicycles in fog, rain, snow, and dust storms. This dataset is more helpful for our study on the problem of recognition of autonomous vehicles in adverse weather. Since the number of motorcycles in this dataset is too small, we discard it and study only the recognition problem of people, cars, buses, trucks and bicycles. The schematic diagram of the adverse weather dataset is shown in Figure 1.

**Figure 2:**
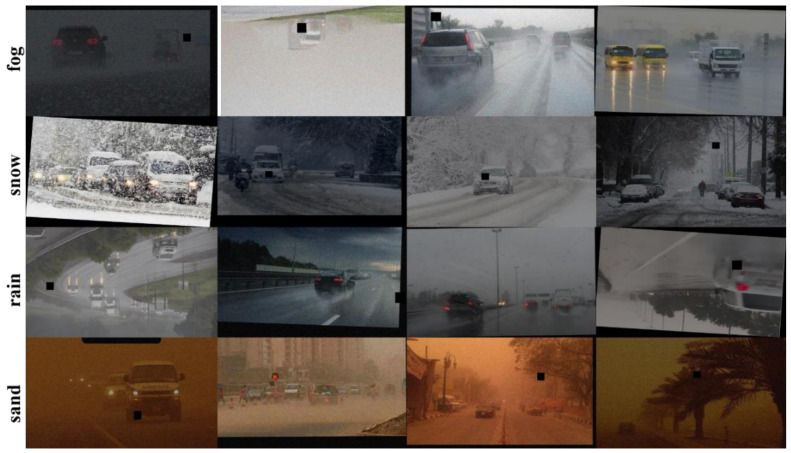
Schematic diagram of the augmented dataset.

Since the DAWN dataset has only 1027 sheets, and the dataset is full of dense small targets with low resolution, the number of datasets is not enough to support the study. Therefore, we perform data enhancement based on the original dataset. We used crop, pan, rotate, mirror, cutout, change brightness and added Gaussian noise to enhance the dataset, and the enhanced dataset is shown in Figure 2. The quality of the dataset is related to the effectiveness of detection and model performance. Increasing the dataset can prevent the phenomenon of overfitting caused by insufficient data during the training process and can improve the robustness and generalization ability of the model. By randomly combining multiple data enhancement methods, the original dataset was expanded by a factor of 4, and 4108 images were acquired, for a total of 5135 images. The breakdown of dataset categories and quantities is shown in Table 1.

### 2.2. Traditional Auto Color Levels Algorithm

The presence of adverse weather can lead to reduced recognition of road targets by self-driving vehicles; thus, we need to design an image processing algorithm to perform filtering on the input side of the image. When the vehicle vision system detects outside information, it first performs image filtering and then passes the completed results of the processing to the target detection algorithm for recognition.

**Figure 3:**
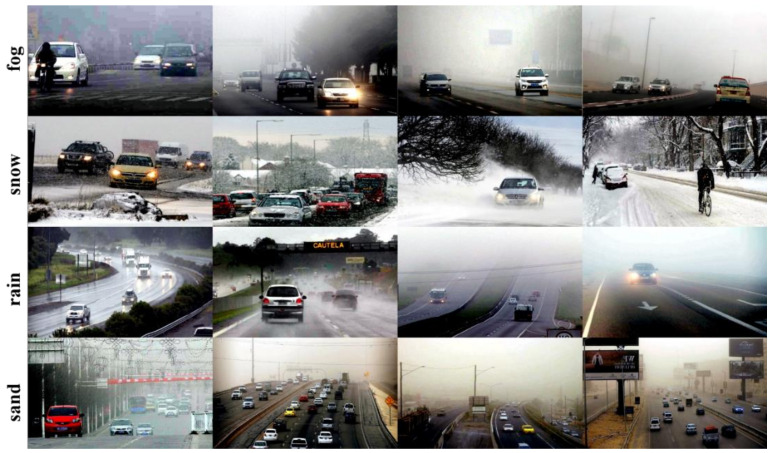
Image after auto color levels algorithm processing.

Auto color levels algorithms are commonly used for image enhancement. In the process of image clarity restoration, the auto color levels algorithm first calculates the pixel grayscale of the image and counts the maximum and minimum grayscale and then divides the pixels in the image into three parts by limiting the maximum and minimum grayscale. If the pixel value is less than the minimum gray value, the pixel gray value is assigned to 0. Conversely, if the pixel gray value is greater than the maximum gray value, the pixel is assigned to 255. If the pixel is in between, the pixel is linearly mapped or gamma corrected to the 0–255 range, and the pixel is automatically restored by normalization; see Equation (1). It was found that the auto color levels algorithm is very effective in removing fog, and it has some enhancement effect when processing other background images. However, the disadvantage is also obvious, and it tends to bring some color distortion when processing other background images [28]. The images processed with the auto color levels algorithm are shown in Figure 3.

(1) $$\left\{\begin{array}{cc} F(u)=0 & u\leq min\\ F(u)=255 & u\geq max\\ F(u)=\left(\frac{u-min}{max-min}\right)\cdot 255 & min<u<max \end{array}\right.$$

### 2.3. Improved Adaptive Color Levels Algorithm

As mentioned above, although there are many people working on filtering algorithms for images, most of them are only for the processing of a specific class of background images. Such algorithms are often not adaptable to a wide range of weather conditions. For example, there is more literature studying the fog removal algorithm, which has achieved some success in the fog removal effect but performs poorly in other severe weather backgrounds. Since self-driving cars need to be adaptive when encountering different weather during motion, we further study based on the previous literature.

Although the auto color levels algorithm can restore image sharpness to some extent, the process is not adjustable, which leads to poor pixel differentiation and some features turning dark black when processing images with different backgrounds. It was found that the contrast stretching algorithm can obtain better filtering effect when processing images with different regions. Therefore, we designed an adaptive color levels algorithm based on the auto color levels algorithm with reference to the idea of contrast stretching algorithm. By constructing a new bias compensation model, the different background images are compensated with differentiation. First, we used the Otsu algorithm to obtain the difference values between image categories separately [29]. Then, we took two groups of images with more obvious differences in the same kind of images and obtained the difference values as light and dark control groups and used the least squares method to obtain the corresponding equation coefficients. Finally, the constructed bias compensation model was used to compensate for the bias of the similar images. The details are shown in Equation (2):

(2) $$\left\{\begin{array}{l} F(u)=\left(\frac{u-min}{max-min}\right)\cdot 255+U\\ U=a\cdot T+b\\ T=\sqrt{\frac{S_{1}+S_{2}+\cdots +S_{n}}{n}} \end{array}\right.$$

where S is the difference value between each image class; n is the number of images; and U is the final bias compensation. The optimal formula is fitted by least squares, and the values of a and b are found.

During the experiment, we fit the four types of background images separately. We first selected two groups of images with more obvious difference values in each class of background images as the light and dark control groups. For each group, eight images with typical features were selected as the dataset for the experiment, i.e., n is set to 8. Since we have obtained a more suitable U value through the experiment in advance, the values of a and b can be easily fitted. Finally, the best U value can be further obtained by the fitted a vs. b. In order to verify whether the fitted a and b are optimal, we first selected six sets of data greater than U and less than U to refit the values of a and b on this basis, where the difference between each set of data is 2. This allowed us to obtain different biases. Then, the images were refiltered using the different biases. Finally, we used these images to compare with the original image and found the corresponding SSIM. By analyzing the trend of SSIM, we could find the most suitable values of a and b. The experiments prove that the SSIM of the latest obtained image is not much different from the first fitting result, which proves that our fitting effect is desirable.

**Figure 4:**
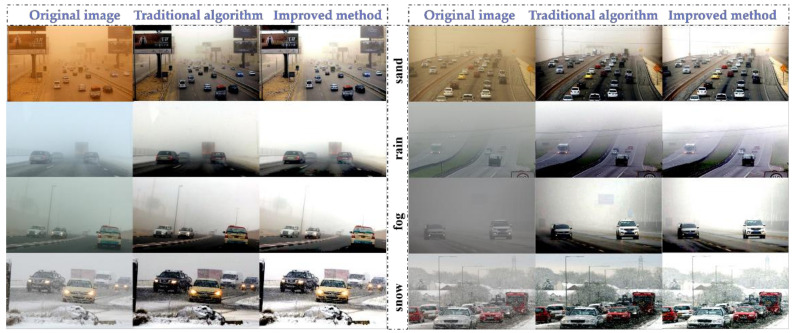
Comparison of before and after improvement of auto color levels algorithm.

The improved adaptive color levels algorithm can control the histogram distribution of the output image more flexibly, adjust the contrast for specific regions of interest, and enhance the image quality. The image effect before and after the improvement is compared with the original image as shown in Figure 4. It can be clearly seen in the figure that the overall effect of the image corrected by the adaptive color scale algorithm is the best and the details are more obvious. The method performs a targeted analysis of fog, rain, snow and sandstorm weather backgrounds, fitting the corresponding bias models and performing the corresponding image processing work. This method solves to a certain extent the challenge of processing images with extensive severe weather backgrounds.

## 3. Method

### 3.1. One-Stage Target Detection Algorithm

Since this research needs to be applied to self-driving vehicles, which require high real-time performance, we choose to improve on the one-stage type algorithm. Currently, the commonly used one-stage algorithms are mainly SSD [30] and YOLO [31]. With the development of YOLO, the most widely used one is YOLOv5. YOLOv5 has a huge improvement in training speed and overall performance compared to the previous versions. YOLOv5 has the characteristics of fast detection, a high recognition rate and more lightweight. Currently, YOLOv5 has several network models, among which YOLOv5s network model has a faster training speed while keeping the network depth and feature map width small, without reducing recognition accuracy too much. The model reduces the computational complexity and is more suitable for porting to embedded devices, which meets our needs.

The CNN of YOLOv5 consists of four main parts, namely Input, Backbone, Neck and Prediction. The input side mainly performs data pre-processing operations, including Mosaic data enhancement, adaptive anchor and adaptive image scaling. Mosaic data enhancement increases the dataset by flipping and stitching to prevent overfitting caused by insufficient data during training. Backbone is mainly used for feature extraction, consisting of Focus, CSP, Conv and C3 modules. Neck draws on PANet’s FPN + PAN structure to pass down high-level semantic information, making features at all scales rich in semantic information. Neck performs multi-scale feature fusion of feature maps at different scales, improving the perceptual field and enriching the expressiveness of feature maps. Prediction is mainly used to predict the type and location of the target, using the features extracted earlier to make predictions.

As an important part of the target detection algorithm, the loss function has the function of measuring the degree of difference between the predicted and true values of the model, which greatly determines the performance of the model. YOLOv5 has three loss functions, namely, classification loss, localization loss, and confidence loss. Classification loss is mainly used to calculate whether the anchor frame is correctly classified with the corresponding calibration; localization loss is used to calculate the error between the prediction frame and the calibration frame, and confidence loss is used to calculate the confidence level of the network [32].

The latest version of YOLOv5 improves DIOU by replacing it with the CIOU loss function. CIOU adds the loss of the detection frame scale to DIOU by increasing the loss of the length and width so that the prediction frame will be more in line with the real frame [33]. The CIOU calculation equations are given in Equations (3)–(6):

(3) $$IOU=\frac{A\cap B}{A\cup B}$$

(4) $$L_{CIOU}=1-CIOU=1-IOU+\frac{\rho^{2}\left(b,b^{gt}\right)}{c^{2}}+\alpha v$$

(5) $$\alpha =\frac{v}{(1-IOU)+v}$$

(6) $$v=\frac{4}{\pi^{2}}{\left(\arctan\frac{w^{gt}}{h^{gt}}-\arctan\frac{w}{h}\right)}^{2}$$

where the parameters A and B denote the area of Ground truth bounding box and the area of predicted bounding box; c denotes the Euclidean distance of the diagonal vertices of the closed box; ρ denotes the Euclidean distance of the center of mass of Ground truth bounding box and predicted bounding box; $\rho^{2}\left(b,b^{gt}\right)$ denotes the distance of the center points of the two boxes; α is the balance parameters, and ν is the indicators to evaluate whether the is an index to evaluate the consistency of the aspect ratio between the Ground truth bounding box and the predicted bounding box. w, h, $w^{gt}$ and $h^{gt}$ represent the height and width of the predicted frame and the real frame, respectively.

### 3.2. Transformer

The transformer is an algorithm designed based on the attention mechanism that facilitates performance improvement [34]. Compared to deep learning models that have residual and convolutional structures, its network structure is simpler and faster in training and inference. The transformer first performs positional encoding of the results of the feature extraction network, recombining the major input vectors to obtain more perfect features. The results are then computed and output in multi-scale parallel by the decoding process. The core formulation is given in Equation (7) [35]. By assigning different weights, more attention can be paid to the feature map. The transformer abandons the structure of RNN and CNN and uses only the attention mechanism, which improves the parallel computing capability of the model and thus the training speed of the model.

(7) $$Attention(Q,K,V)=softmax(\frac{QK^{T}}{\sqrt{d_{k}}})V$$

Q,K,V represent Query vector, Key vector and Value vector, respectively. For gradient stabilization, Transformer uses score normalization, i.e., dividing by $\sqrt{d_{k}}$.

We can understand the Transformer as a black box; when we perform the image recognition task, the input information passes through this black box to extract the deep features. The structure of the Encoder is an attention mechanism plus a feed-forward neural network, which scores the input data and then normalizes the score by softmax operation to extract the feature information accurately. The Encoder has six encoders, and the Decoder has six decoders. This design can speed up the processing of information by parallel operation.

### 3.3. Attention Model

Due to the good performance of attentional mechanisms, attentional mechanisms are commonly added in current research to achieve performance improvement. The most commonly used attention mechanisms are SE, CAM, SAM, and CBAM, among others. The attention mechanism transforms the input into a single feature vector and obtains significant performance gains with very little computational cost, which can focus on important features and suppress unnecessary features [31]. Convolutional operations extract information features by mixing cross-channel and spatial information, while CBAM can emphasize meaningful features in two main dimensions, the channel axis and the spatial axis. Attentional mechanisms improve accuracy by focusing on important features and suppressing unnecessary features [36].

CBAM is actually a serial structure of CAM and SAM, combining the advantages of both attention mechanisms, which can fully improve the representational power of CNN. CAM generates channel attention graphs using the channel relationship between features and uses the method of compressing the spatial dimension of input feature mapping to calculate channel attention. CAM uses a combination of AvgPool and MaxPool, which has more representational power; see Equation (8). SAM only considers the optimization of the feature map in the spatial (Spatial) dimension, with maximum pooling and average pooling in the spatial dimension, respectively, to provide more attention to task-relevant regions; see Equation (9). The specific operation of CBAM is formulated in Equations (10) and (11) [36].

(8) $$\begin{array}{l} M_{c}\left(F\right)=\sigma \left(MLP\left(AvgPool\left(F\right)\right)+MLP\left(MaxPool\left(F\right)\right)\right)\;\\ \;\;\;=\sigma \left(W_{1}\left(W_{0}\left(F_{avg}^{{}^{c}}\;\right)\right)+W_{1}\left(W_{0}\left(F_{max}^{{}^{{}^{c}}}\;\right)\right)\right) \end{array}$$

(9) $$\begin{array}{l} M_{s}\left(F\right)=\sigma \left(f^{7\times 7}\;\left(\left[AvgPool\left(F\right);MaxPool\left(F\right)\right]\right)\right)\;\\ \;\;\;=\sigma \left(f^{7\times 7}\;\left(\left[F_{avg}^{{}^{s}};\;F_{max}^{{}^{s}}\right]\right)\right) \end{array}$$

(10) $$F^{\prime }=M_{c}\left(F\right)\;⊗\;F$$

(11) $$F^{\prime \prime }=M_{s}\left(F^{\prime }\right)\;⊗\;F^{\prime }$$

where σ denotes the sigmoid function; $W_{0}\in R^{C/r\times C}$ and $W_{1}\in R^{C\times C/r}$;$f^{7\times 7}$ represents a convolution operation with the filter size of 7 × 7; F represents the Feature Map, $F^{\prime }$ is the channel attention output; the CBAM output $F^{\prime \prime }$ is obtained after computing the channel attention output $F^{\prime }$ with the spatial attention weights.

### 3.4. Proposed Methodology

By studying the previous methods, we found that the improvement based on YOLOv5 has achieved good results. Although Transformer and CBAM have been widely used, there are few cases where Transformer, CBAM and EIOU are applied to the project at the same time. By adopting their advantages in one, we find that their detection effect is outstanding, not only in detecting different scale targets, but also in extracting deep features with obvious advantages.

We first dynamically adjust the number of Backbone parameters of the original CNN of YOLOv5 to increase the model complexity and improve the network by adding Transformer and CBAM modules [9,10]. Then, the loss function of EIOU is used to replace the loss function of the original CIOU to make up for the deficiency of CIOU itself and further improve the accuracy of the model [37]. Finally, the K-means++ clustering algorithm is used to automatically cluster the labeled target bounding anchor boxes in the dataset to produce different numbers and sizes of a priori boxes. This approach can increase the matching degree between the a priori frames and the actual target frames, thus further improving the detection accuracy. The specific details are as follows.

**Figure 5:**
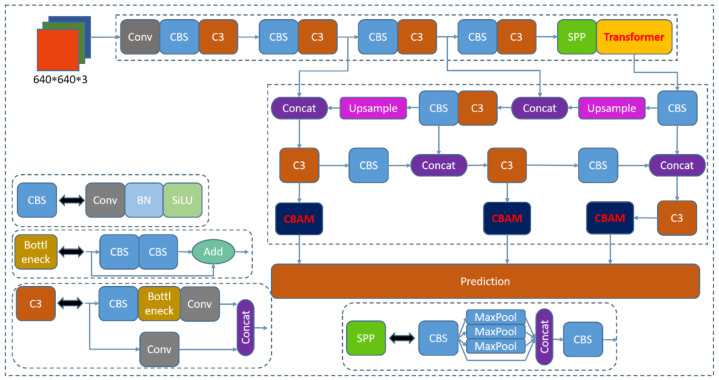
Improved YOLOv5 network structure diagram.

We add Transformer and CBAM, respectively, to the CNN of YOLOv5 and improve the CNN by ablation experiments. We add the Transformer module at the tail of the feature extraction network to make it adjacent to the SPP module. This design allows the Transformer to process the features extracted by the CNN more adequately and enhance the global nature of the network feature extraction. The Transformer can divide the deep semantic features into multiple branches and assign weights to the feature perceptual fields to extract key features and enhance the semantic representation on multiple scales [38]. It was found that by adding Transformer to the feature extraction network, image features can be extracted efficiently. This can improve the parallel computing capability of the model to a certain extent, which in turn improves the training speed and recognition accuracy of the model. Meanwhile, we add a CBAM module to each prediction channel before the output side. This method allows CBAM to further focus on the features extracted by the C3 module and further enhance the attention of the perceptual field. In the third channel, two deep feature extraction modules, Transformer and CBAM, exist, and this channel mainly predicts the targets with smaller size. Since the effective features extracted by Transformer and CBAM are relatively focused and complete [38], this pathway is beneficial to improve the detection accuracy of dense small targets. The other two pathways also improve the recognition accuracy for large targets as well as medium targets due to the presence of the CBAM module. The network structure diagram is shown in Figure 5.

Although CIOU Loss takes into account the overlap area, centroid distance, and aspect ratio of the bounding box regression, Equation (4) reflects the difference in aspect ratio rather than the true difference between the width and height and their confidence levels; thus, it sometimes prevents the model from optimizing the similarity effectively [37]. The penalty term of EIOU is based on the penalty term of CIOU by splitting the influence factor of the aspect ratio to calculate the length and width of target and anchor frames separately. The loss function contains three components: overlap loss, center distance loss, and width–height loss. The first two parts continue the method in CIOU, but the width–height loss directly minimizes the difference between the width and height of the target box and the anchor box, which makes the convergence faster. The formula for the penalty term is given in Equation (12) [37].

(12) $$L_{EIOU}=L_{IOU}+L_{dis}+L_{asp}=1-IOU+\frac{\rho^{2}\left(b,b^{gt}\right)}{c^{2}}+\frac{\rho^{2}\left(w,w^{gt}\right)}{C_{w}^{2}}+\frac{\rho^{2}\left(h,h^{gt}\right)}{C_{h}^{2}}$$

where $C_{w}$ and $C_{h}$ are the width and height of the smallest enclosing box covering the two boxes. Namely, we divide the loss function into three parts: the IOU loss $L_{IOU}$, the distance loss $L_{dis}$ and the aspect loss $L_{asp}$. C represents the area of the Ground truth bounding box and the minimum enclosing box of the predicted bounding box [37].

We have achieved good results by adding Transformer and CBAM to the original CNN of YOLOv5. In order to further improve the performance of the model, we replaced the original CIOU loss function with the more accurate EIOU at the output side and found that the replacement of the loss function can make the model more accurate in regression frame localization and improve the detection performance of the model. In particular, the model performs better when there are multiple targets in close proximity or when the size of the immediate targets varies greatly. In this case, the EIOU loss function calculates the length and width of the target frame and anchor frame separately, which makes the width and height loss closer to the actual size, thus further improving the recognition rate of the model.

The above improvement method improves the complexity of the original CNN and enhances the feature extraction ability of the network. By continuously delivering clear target features to the deep network, the problem of weak target recognition in bad weather is solved. Meanwhile, Transformer and CBAM modules are introduced to solve the problem of invalid features affecting recognition accuracy and mitigate the impact of bad weather on the target detection algorithm. The robustness of the model is further enhanced by replacing the loss function as EIOU. The algorithm pseudo-code is shown in Table 2.

## 4. Experimental Results and Analysis

### 4.1. Evaluation Criterion

Image noise is generally evaluated using MSE, PSNR and SSIM [39]. MSE is a more convenient way to measure the mean error, and MSE can evaluate the degree of variability of the data. The smaller the value of MSE, the better the accuracy of the prediction model in describing the experimental data. To measure the quality of the processed image, we usually refer to the PSNR value to measure whether the filtering algorithm is satisfactory or not; a higher PSNR value means less distortion. SSIM is a measure of the similarity of two images. One of the two images used in SSIM is an uncompressed undistorted image, and the other is a distorted image. The range of SSIM is from 0 to 1. The value of SSIM is equal to 1 when the two images are identical. As an implementation of SSIM theory, the SSIM index defines structural information from the perspective of image composition as independent of luminance and contrast and reflects the properties of object structure in the scene and models distortion as a combination of three different factors: luminance, contrast, and structure. The mean value is used as an estimate of luminance, the standard deviation as an estimate of contrast, and the covariance as a measure of structural similarity, as specified in Equation (13).

(13) $$\left\{\begin{array}{l} MSE=\frac{1}{N}{\sum_{i}^{N}\left(x_{i}-y_{i}\right)}^{2}\\ PSNR=10\log_{10}\frac{{\left(2^{r}-1\right)}^{2}}{MSE}=20\log_{10}\frac{2^{r}-1}{MSE}\\ SSIM=\frac{\left(2\mu_{x}\mu_{y}+c_{1}\right)\left(O_{xy}+c_{2}\right)}{\left(\mu_{x}^{2}+\mu_{y}^{2}+c_{1}\right)\left(O_{x}^{2}+O_{y}^{2}+c_{2}\right)} \end{array}\right.$$

We name the original image as X and the filtered image as Y, where the elements are noted as $x_{i}$ and $y_{i}$; r is the number of bits of each sampled value; $\mu_{x}$ is the mean of x, $\mu_{y}$ is the average of y; $O_{x}^{2}$ is the variance of x, $O_{y}^{2}$ is the variance of y; $O_{xy}$ is the covariance of x and y; $c_{1}={\left(k_{1}L\right)}^{2}$ and $c_{2}={\left(k_{2}L\right)}^{2}$ is the constant to maintain stability; $k_{1}$= 0.01, $k_{2}$= 0.03; L is the dynamic range of pixel values, generally L = 255.

In this paper, we use Precision, Recall, F1-score, mAP and FPS as evaluation metrics, and visualize and compare different algorithms using PR curves to analyze the performance of different models. Precision represents the percentage of correctly predicted results among all predicted positive samples. Recall represents the percentage of correctly predicted results among all positive samples. F1-Score is used to measure the precision of the dichotomous classification model, taking into account both the precision and recall of the classification model. mAP represents the mean value of the precision obtained at different recall rates, as shown in Equation (14). As we can see from the formula, AP is the integral of the PR curve, that is, the area. The PR curve represents the relationship between precision and recall, and generally we believe that the larger the area of the PR curve, the better the model performance. mAP@0.5:0.95 denotes the mean average precision at different IOU thresholds (IOU from 0.5 to 0.95 in steps of 0.05). The detection speed is generally measured by FPS, which indicates the number of images that the target detection network can process per second, and the larger the value of FPS, the faster the network model can process images [40,41]:

(14) $$\left\{\begin{array}{l} P=\frac{TP}{TP+FP}\\ R=\frac{TP}{TP+FN}\\ F1=2\cdot \frac{P\cdot R}{P+R}\\ AP=\overset{m}{\underset{i=1}{\sum }}P\left(i\right)\Delta R\left(i\right)=\int_{0}^{1}P\left(R\right)dR\\ mAP=\frac{\sum_{m=1}^{M}AP\left(n\right)}{M} \end{array}\right.$$

where TP is the positive sample predicted by the model as a positive category; FP is the negative sample predicted by the model as a positive category; and FN is the positive sample predicted by the model as a negative category. m in the formula denotes the category, and M denotes the total number of categories.

### 4.2. Image Effect Evaluation and Analysis

In order to effectively evaluate the difference between the improved adaptive color levels algorithm and the traditional auto color levels algorithm, three evaluation indexes, MSE, PSNR and SSIM, are chosen to compare the original image and the image processed by the traditional method in Figure 4 with the true value. Since the DAWN dataset does not provide true values, the analysis in Figure 4 shows that the images processed by the adaptive color levels algorithm proposed in this paper have better results; thus, we choose such maps as the estimated true values. The experimental results are shown in Table 3, from which it can be clearly seen that the difference in MSE values between the original image and the estimated true value is larger in the case of sandstorm, rain and fog, which indicates that the difference from the original image to the true value is greater. Similarly, it can be seen that the PSNR and SSIM between the traditional method and the estimated true value are relatively small, which indicates that the proposed method obtains a better improvement than the traditional method. However, the MSE between the traditional method and the estimated true value is larger than the MSE between the original and the estimated true value in snowy weather. Furthermore, the PSNR between the traditional method and the estimated true value is lower than the PSNR between the original image and the estimated true value, and the details of the image become much darker after the traditional auto color levels algorithm, which is different from the original image, resulting in a lower PSNR index. At the same time, the reason for this result may also be related to the absence of the true value image, and there is still some difference between the image processed by the improved method of this paper as the estimated true value and the actual effect, but in general, it can show that the improved adaptive color levels algorithm has good effect on poor image optimization.

### 4.3. Algorithm Comparison and Quantitative Analysis

Since the algorithm is mainly used in self-driving vehicles, which require high real-time performance, we conducted comparison tests under the same conditions for the commonly used one-stage algorithm. One-stage algorithms mainly include SSD and YOLO algorithms, and we chose the corresponding tiny version for the YOLO algorithm. We finally compared the performance metrics of SSD, YOLOv3-tiny, YOLOv4-tiny, YOLOv4-mobileNetv2, YOLOv4-mobileNetv3, YOLOv4-ghostNet and YOLOv5 with the same dataset.

In the training process, we divided the dataset into training set, validation set and test set in the ratio of 7:2:1. The K-means++ algorithm is used to recluster the dataset before training to automatically obtain the best anchor size to replace the anchor box size in the original COCO dataset. In the training process of YOLOv5, we chose YOLOv5s as the pre-training model for YOLOv5, which reduces the time to train a large number of datasets and improves performance. We also used the warm-up approach to pre-warm up the learning rate. In the warm-up phase, the learning rate is updated using one-dimensional linear interpolation, and the learning rate is updated using cosine annealing at the end of the warm-up [42]. The main development platforms and hyperparameters information are shown in Table 4.

To make the experimental comparison more rigorous, we first tested the original dataset using the above algorithm. In Table 5, we can see that SSD, YOLOv3-tiny, YOLOv4-tiny, YOLOv4-mobileNetv2, YOLOv4-mobileNetv3 and YOLOv4-ghostNet all have lower mAP when the IOU is 0.5 or 0.75. In contrast, the YOLOv5 algorithm achieves better results.

We used the adaptive color levels algorithm to enhance the original dataset to obtain images with clearer backgrounds. Then, the enhanced dataset was retrained again using the above algorithm, and the specific data are shown in Table 6. It can be seen in the data that the recognition accuracy of the dataset after image processing was improved to some extent, but the overall performance of SSD, YOLOv3-tiny, YOLOv4-tiny and their corresponding lightweight models still failed to meet our requirements. On the contrary, the model performance of the YOLOv5 algorithm still performs well, which proves the superiority of the YOLOv5 algorithm. It also shows that the relatively deep feature extraction network plays a crucial role in the process of target mention detection in bad weather. Therefore, it is necessary for us to improve the YOLOv5 algorithm.

During the improvement of the YOLOv5 algorithm, we mainly made parameter adjustments in the original CNN as well as add modules such as SE, CBAM and Transformer. To better improve the network, we selected and optimized the algorithm utilizing ablation experiments, as shown in Table 7. By continuously comparing the positions of tuning SE, CBAM and Transformer and the parameter values, we found that adding the Transformer and CBAM to the original feature extraction network and modifying the loss function to EIOU had the greatest effect on the improvement of detection accuracy.

Since the target detection task in the field of autonomous driving requires faster computing speed, we chose the algorithm with better effect of one-stage for comparison experiments and quantitatively evaluated the improved YOLOv5 algorithm before and after. In order to verify the effectiveness of the proposed algorithm, we compared it with some lightweight detection algorithms and mainstream one-stage target detection algorithms, and the experimental results are shown in Table 5, Table 6 and Table 7. In Table 5, it can be seen that the YOLOv5 algorithm performs the best among the commonly used one-stage algorithms with a mAP of 84.7 percentage points, which is the reason why we choose to improve on this basis. In Table 6, it can be seen that the original YOLOv5 algorithm performs well in the enhanced dataset, with a mAP of 92.9 percentage points. In Table 7, we can see that the improved YOLOv5 algorithm achieves a mAP of 94.7 percentage points in the enhanced dataset, which is another 1.8 percentage points improvement compared to the previous one. By analyzing the differences between the two algorithms, we can see that the original YOLOv5 algorithm performs well but does not add the attention mechanism, while the improved algorithm incorporates the Transformer and CBAM modules, resulting in a further improvement in overall performance. In the “B-T” experiments in Table 7, it can be seen that by adding the Transformer mAP improved by 0.5 percentage points compared to 92.9 percentage points before the improvement. Similarly, in the “B-CBAM” experiment, it can be seen that by adding CBAM, the overall mAP also increases by 0.5 percentage points. This shows that adding Transformer and CBAM to the YOLOv5 algorithm helps to improve the recognition accuracy. In addition, we compared the performance parameters when the loss functions were CIOU and EIOU after adding Transformer and CBAM, respectively. The results show that the mAP improves again when the improved algorithm selects EIOU as the loss function. In general, YOLOv5-improve performs better among many models in the image recognition task for severe weather.

### 4.4. Visualization Comparison and Analysis

**Figure 6:**
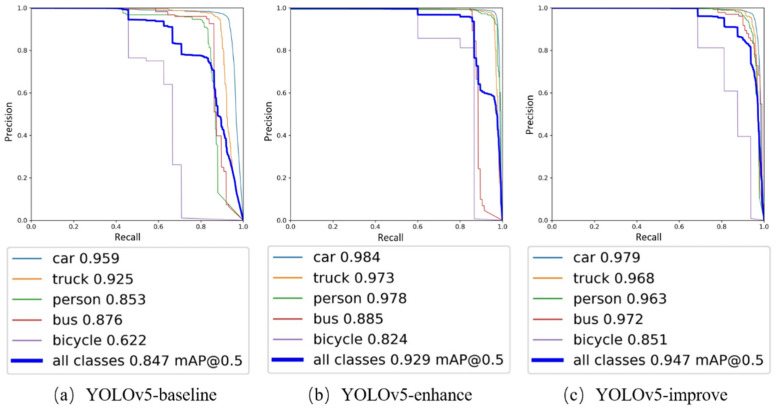
YOLOv5 before and after the improvement of the PR curve comparison chart.

The corresponding PR curves were obtained using the improved YOLOv5 algorithm before and after training on the original and enhanced datasets. The PR curves are mainly the area composed of P and R. In Figure 6, it can be clearly seen that the area of the three plots in general becomes larger in turn. Meanwhile, analyzing the AP and mAP of the three algorithms also shows that YOLOv5-improve has a 10% improvement compared to the mAP of YOLOv5-baseline. It can be concluded that the improved YOLOv5-improve algorithm has the best recognition effect on the severe weather images processed by the adaptive color scale algorithm, and the target recognition accuracy is greatly improved.

**Figure 7:**
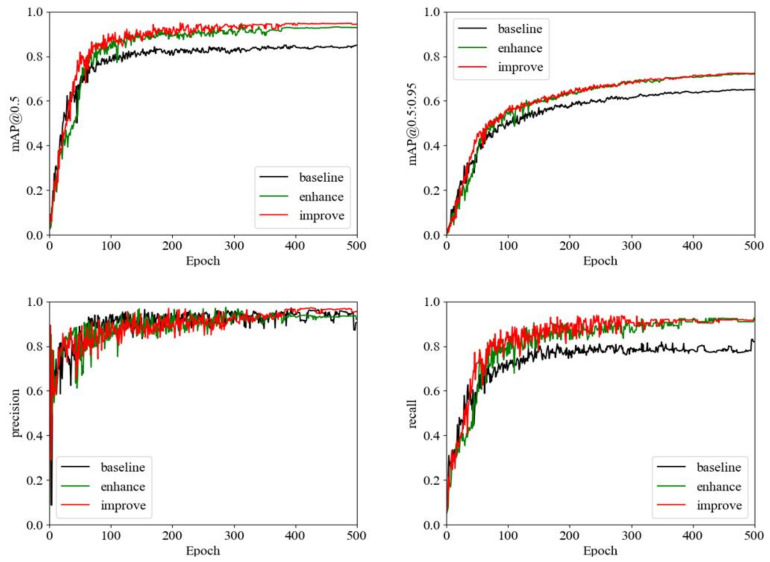
Comparison chart of training curve visualization.

We used TensorBoard to monitor the metric curves of the model training data (mAP@0.5, mAP@0.5:0.95, Precision, Recall) in real time during the model training, and we performed a real-time visual inspection of the main performance metrics of the algorithm before and after the improvement, as shown in Figure 7. With the increase in epoch, the model performance metrics keep changing. Among them, mAP@0.5, Precision and Recall basically level off at epoch of 400, but the mAP@0.5:0.95 index still has an increasing trend. To extract as many features as possible and achieve better performance, we finally set Epoch to 500 for comprehensive consideration. Compared with normal images, it is more difficult to train images in bad weather, and in Figure 7, we can see that the YOLOv5-improve algorithm has the best results in all four metrics, which also proves the superiority of our improved algorithm.

### 4.5. Comparison of Detection Effects and Qualitative Analysis

The recognition of the image targets in adverse weather is a more difficult problem that poses a challenge in the field of computer vision. Specifically, the background of bad weather poses difficulties for target detection. In this case, targets of various scales (humans, bicycles, cars and trucks, etc.) are equally difficult to detect, which further cause a reduction in recognition accuracy [16].

**Figure 8:**
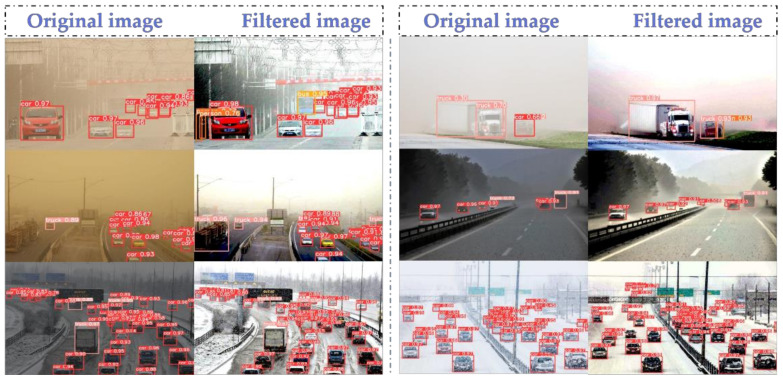
Graph of recognition results before and after image filtering.

We qualitatively evaluated the detection effect of YOLOv5-baseline and YOLOv5-improve using six sets of images of the scene. We used the same parameters to detect the images, and the experimental results are shown in Figure 8. In the detection results of the original image, it can be seen that there are problems of missed detection, false detection or low recognition rate for large-sized trucks and small-sized humans. However, the YOLOv5-improve algorithm has significantly better detection results for the filtered images, making up for the shortcomings of the original algorithm.

Analyzing the reasons, we can see that Transformer has a self-attention mechanism, which can effectively obtain global information, and multiple heads can map it to multiple spaces, making the model expressive. CBAM focuses attention on important points among many information points, selecting key information and ignoring other unimportant information. We have extracted the depth of object features by adding Transformer with the CBAM module, which is beneficial to the detection effect of the final target. At the same time, replacing the loss function with a more accurate EIOU is also beneficial to the recognition rate. Finally, the algorithm is more accurate for feature extraction and is more adaptable to multi-size image detection. Overall, the images processed by the adaptive color scale algorithm are more easily recognized by the improved YOLOv5-improve algorithm.

### 4.6. Failure Case Study

**Figure 9:**
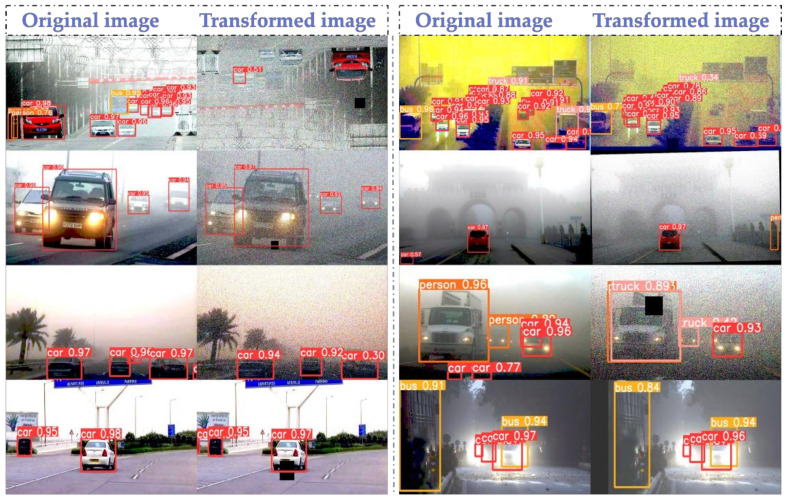
Comparison of test results of failed cases.

During the test, we found that the original image processed by the adaptive color levels algorithm has good results, but there are also problems of recognition errors in special cases, as shown in Figure 9. For example, the morphologically transformed images have problems such as flip and noise, resulting in missed detection and low recognition rate. At the same time, due to the low resolution of the original image, after the image processing, some details are not processed well, which also causes the occurrence of false detection and low recognition rate.

## 5. Conclusions

In summary, we focused on the problem of how to improve the recognition rate of self-driving vehicles for road targets in adverse weather conditions. In this paper, we first used Gaussian noise addition, rotation angle adjustment, mirroring and cutout to pre-process the dataset to increase the number and diversity of the dataset. Then, a new bias compensation model was constructed to perform adaptive color levels correction on the images. The algorithm can automatically assign preprocessing channels according to different types of input images, and perform rain removal, fog removal, snow removal and dust storm removal operations on the images. Finally, the algorithm was improved by adding Transformer and CBAM modules to the original YOLOv5 algorithm, while the loss function of YOLOv5 was changed to the EIOU function. By comparing SSD, YOLOv3-tiny, YOLOv4-tiny, YOLOv5-baseline, YOLOv5-enhance and YOLOv5-improve, it is obvious in the evaluation index comparison table and PR curve comparison graph that the improved model of this paper has the best performance with mAP reaching 94.7% and FPS of 199.86. In Figure 8, it can also be clearly seen that the recognition capability of the improved algorithm is greatly improved under the severe weather images processed by the adaptive color levels algorithm. Overall, the research content of this paper has improved the recognition ability of self-driving cars in bad weather to a certain extent and achieved the research objective.

## Figures and Tables

**Table 1:** Breakdown of dataset categories and quantities.

Classes	Samples
Rain	1000
Snow	1020
Fog	1500
Sandstorm	1615
Total	5135

**Table 2:** Algorithm pseudo-code.

Preliminary Work: Data Widening, Expanding the Number of Datasets and Preventing Overfitting.
1. Input dataset; 2. An improved adaptive color levels compensation algorithm is used to obtain a series of clear images as follows: $\left\{\begin{array}{cc} F(u)=0 & u\leq min\\ F(u)=255 & u\geq max\\ F(u)=\left(\frac{u-min}{max-min}\right)\cdot 255+U & min<u<max \end{array}\right.$ 3. The filtered dataset is passed to the modified YOLOv5 for training, and the final model is trained by adjusting the Loss through EIOU. The EIOU equation is as follows: $L_{EIOU}=L_{IOU}+L_{dis}+L_{asp}=1-IOU+\frac{\rho^{2}\left(b,b^{gt}\right)}{c^{2}}+\frac{\rho^{2}\left(w,w^{gt}\right)}{C_{w}^{2}}+\frac{\rho^{2}\left(h,h^{gt}\right)}{C_{h}^{2}}$ 4. Combined with the trained network model, the region of interest of the input image is accurately and quickly calculated to obtain the target class.
Output: target recognition results.

**Table 3:** Results of evaluation metrics between different images and estimated true values.

	Indicator	MSE		PSNR		SSIM	
Classes		Comparison Results between the Original and Estimated True Values	Comparison Results of Traditional Method and Estimated True Value	Comparison Results between the Original and Estimated True Values	Comparison Results of Traditional Method and Estimated True Value	Comparison Results between the Original and Estimated True Values	Comparison Results of Traditional Method and Estimated True Value
Sand		3785.98	1934.95	12.35	15.26	0.74	0.85
		3331.59	1709.31	12.90	15.80	0.77	0.84
Rain		2187.94	1140.79	14.73	17.56	0.86	0.87
		3772.33	1696.24	12.36	15.84	0.81	0.89
Fog		3779.89	1673.98	12.36	15.89	0.85	0.88
		8361.95	1809.93	8.91	15.55	0.69	0.82
Snow		1890.27	2448.35	15.37	14.24	0.79	0.84
		1845.99	2358.05	15.47	14.41	0.82	0.83

**Table 4:** Development platform information.

Category	Name	Parameters
Hardware configuration	CPU GPU Memory	I7-11700F RTX3060 Dual Graphics Card 32G
Software configuration	Operating System Frameworks Python CUDA	Ubuntu20.04 Pytorch 3.8 11.1
Hyperparameter setting	Input image Epoch Batch Size Initial learning rate Cyclic learning rate momentum Weight decay	640 × 640 500 64 0.01 0.1 0.937 0.0005

**Table 5:** Comparison of evaluation indicators under the original dataset.

Methods	IOU	Precision	Recall	F1	mAP	FPS
SSD	0.5	0.91	0.24	0.39	37.54%	87.91
	0.75	0.60	0.19	0.29	23.28%	
YOLOv3-tiny	0.5	0.80	0.59	0.68	51.29%	169.03
	0.75	0.50	0.37	0.43	25.37%	
YOLOv4-tiny	0.5	0.82	0.52	0.64	49.90%	207.21
	0.75	0.55	0.35	0.43	29.05%	
YOLOv4-mobileNetv2	0.5	0.83	0.36	0.50	46.46%	86.20
	0.75	0.59	0.25	0.35	22.66%	
YOLOv4-mobileNetv3	0.5	0.80	0.28	0.42	40.48%	73.39
	0.75	0.53	0.19	0.27	16.72%	
YOLOv4-ghostNet	0.5	0.77	0.28	0.41	35.95%	63.12
	0.75	0.49	0.17	0.26	14.82%	
YOLOv5-baseline	0.5	0.91	0.82	0.90	mAP@.5: 84.7%	199.74
	0.5:0.95				mAP@.5:.95: 65.1%	

**Table 6:** Comparison of evaluation indexes after image filtering.

Methods	IOU	Precision	Recall	F1	mAP	FPS
SSD	0.5	0.90	0.27	0.42	39.79%	84.97
	0.75	0.81	0.25	0.38	26.33%	
YOLOv3-tiny	0.5	0.81	0.57	0.67	59.26%	159.82
	0.75	0.49	0.35	0.41	35.36%	
YOLOv4-tiny	0.5	0.92	0.56	0.70	53.57%	209.13
	0.75	0.76	0.47	0.58	37.30%	
YOLOv4-mobileNetv2	0.5	0.85	0.47	0.60	51.32%	90.77
	0.75	0.61	0.27	0.38	23.74%	
YOLOv4-mobileNetv3	0.5	0.82	0.32	0.46	44.21%	76.60
	0.75	0.54	0.21	0.31	18.57%	
YOLOv4-ghostNet	0.5	0.80	0.28	0.42	37.35%	63.41
	0.75	0.52	0.19	0.27	15.70%	
YOLOv5- enhance	0.5	0.94	0.91	0.92	mAP@.5: 92.9%	200.33
	0.5:0.95				mAP@.5:.95: 72.4%	

**Table 7:** Trick ablation experiment.

Methods	IOU	Precision	Recall	F1	mAP@.5	FPS
					mAP@.5:.95	
B-SE	0.5	0.96	0.87	0.91	93.2%	198.73
	0.5:0.95				64.8%	
B-CBAM	0.5	0.95	0.93	0.94	93.4%	206.39
	0.5:0.95				72.9%	
B-T	0.5	0.97	0.91	0.94	93.4%	197.62
	0.5:0.95				73.8%	
B-CBAM-N-CBAM	0.5	0.94	0.91	0.92	92.9%	197.33
	0.5:0.95				72.4%	
B-T-N-SE	0.5	0.94	0.92	0.93	93.3%	202.21
	0.5:0.95				71.4%	
B-T-N-CBAM	0.5	0.93	0.93	0.93	94.1%	200.35
	0.5:0.95				72.2%	
B-T(5)-N-CBAM (CIOU)	0.5	0.90	0.92	0.91	94.3%	201.03
	0.5:0.95				71.0%	
B-T(5)-N-CBAM (EIOU)	0.5	0.97	0.92	0.94	94.7%	199.86
	0.5:0.95				72.3%	

B stands for Backbone, N stands for Neck, T stands for Transformer, and T(5) stands for Transformer parameter set to 5. We name the improved algorithm YOLOv5-improve.

## Data Availability

The data used to support the findings of this study are included within the article.

## Abbreviations

SVM	Support vector machine
UAV	Unmanned aerial vehicle
CNN	Convolutional neural network
mAP	Mean average precision
MSE	Mean Square Error
PSNR	Peak Signal to Noise Ratio
SSIM	Structural Similarity
CAM	Channel attention module
SE	Squeeze and Excitation
PR	Precision–recall
SSD	Single-shot detection
